# Development of NIR-Based ANN Models for *On-Line* Monitoring of Glycerol Concentration during Biodiesel Production in a Microreactor

**DOI:** 10.3390/mi13101590

**Published:** 2022-09-25

**Authors:** Martin Gojun, Davor Valinger, Anita Šalić, Bruno Zelić

**Affiliations:** 1 Deptartment of Reaction Engineering and Catalysis, Faculty of Chemical Engineering and Technology, University of Zagreb, Marulićev trg 19, HR-10000 Zagreb, Croatia; 2 Laboratory for Measurement, Control and Automatisation, Faculty of Food Technology and Biotechnology, University of Zagreb, Pierottijeva 6, HR-10000 Zagreb, Croatia; 3 Department of Thermodynamics, Mechanical Engineering and Energy, Faculty of Chemical Engineering and Technology, University of Zagreb, Marulićev trg 19, HR-10000 Zagreb, Croatia; 4 Department of Packaging, Recycling and Environmental Protection, University North, Trg dr. Žarka Dolinara 1, HR-48000 Koprivnica, Croatia

**Keywords:** biodiesel, glycerol, near infrared spectroscopy, artificial neural networks, *on-line* measurements

## Abstract

During the production process, a whole range of analytical methods must be developed to monitor the quality of production and the desired product(s). Most of those methods belong to the group of *off-line* monitoring methods and are usually recognized as costly and long-term. In contrast, *on-line* monitoring methods are fast, reliable, simple, and repeatable. The main objective of this study was to compare different methods for monitoring total glycerol concentration as one of the indicators of process efficiency during biodiesel production in a batch reactor and in a microreactor. During the biodiesel production process, the glycerol concentration was measured *off-line* using standard methods based on UV-VIS spectrophotometry and gas chromatography. Neither method provided satisfactory results, namely, both analyses showed significant deviations from the theoretical value of glycerol concentration. Therefore, near infrared spectroscopy (NIR) analysis was performed as an alternative analytical method. The analysis using NIR spectroscopy was performed in two ways: *off-line*, using a sample collected during the transesterification process, and *on-line* by the continuous measurement of glycerol concentration in a rector. Obtained results showed a great NIR application potential not only for *off-line* but also for *on-line* monitoring of the biodiesel production process.

## 1. Introduction

Biodiesel, a representative of renewable fuels, can be produced from various substrates, such as vegetable oils, animal fats, and waste cooking oils [1,2]. The diversity of production processes (blending, micro-emulsification, pyrolysis, and transesterification) [3], provides enough methods to explore different operation set-ups. However, the existing conventional processes are hardly able to satisfy the increasing demand for biodiesel on the market. Various methods have been developed for the intensification of biodiesel production processes. The use of microreactors, micro-scale reactor systems characterized by efficient heat and energy transfer in combination with short residence time, allows for a great intensification of the biodiesel production process [4]. Furthermore, the produced biodiesel must meet the required quality standards according to EN 14214 [5]. Therefore, a whole range of analytical methods has been developed to determine the quality of the produced biodiesel. Most of the analytical methods developed for measuring biodiesel quality belong to the group of *off-line* monitoring methods, such as Fourier transform infrared spectroscopy (FTIR) [6,7], high performance liquid chromatography (HPLC) [8], and gas chromatography (GC) [9,10]. The major drawbacks of these methods are the high cost and time required for analysis, both for the method itself and for obtaining and preparing the sample. Moreover, these methods require the use of solvents for sample preparation. Therefore, *on-line* monitoring methods, such as NIR spectroscopy [11,12,13,14], fluorescence [15] or UV spectroscopy [16], and mid-infrared (MIR) spectroscopy [17,18], are used as fast (each analysis requires less than two minutes) and reliable monitoring methods. All of those methods are simple and repeatable, economically justified (since no sample pretreatment is required), and no additional waste is generated [19]. However, to obtain satisfactory results with these methods, a large amount of spectral data must be collected from the experiments. Computer analysis (i.e., principal component analysis (PCA), partial least squares modeling (PLS), artificial neural networks (ANN) etc.) is usually required to identify similarities and differences between samples, since there is considerable spectral overlap [20]. Although there are papers related to biodiesel fuel quality monitoring by NIR spectroscopy using PLS models and also PLS regression models using NIR spectroscopy for *on-line* monitoring of the biodiesel production reaction [21,22], this work was based on ANN because our previous experience with NIR spectroscopy in different fields using linear, non-linear, PLS and ANN models [23,24] has shown better performance of ANN in comparison to other methods. The main problem is the software commonly used for computer analysis (Statistica, MatLab, Unscrambler, or others), which is not able to provide results from raw NIR spectra in a few seconds, since a combination of PCA and PLS or ANN is required. To implement “real” *on-line* monitoring that could potentially be used in the industry, the software solution requires calibration to a reference method that must be applied to develop a robust calibration equation for component monitoring [25].

As mentioned earlier, the quality of the produced biodiesel can be monitored in different ways during production. Some of the methods used are monitoring of mono-, di-, and triacylglycerides, alcohols formation [26], and water content [27] etc., but the two most common methods are monitoring of free fatty methyl esters (FAME) and glycerol formation [28,29], respectively.

The main objective of this study was to compare several methods for monitoring the total glycerol concentration during the biodiesel production process. Since most of the known methods for monitoring biodiesel quality during production are *off-line* and do not provide the necessary information in time, that approach is not satisfactory, especially in continuous biodiesel production. Therefore, NIR spectroscopy in combination with ANNs was used as an alternative method for the analysis of glycerol concentration during biodiesel production in a batch reactor and in a microreactor.

Special attention was paid to the development of a calibration model based on GC analysis, as a reference method for *on-line* monitoring of glycerol by NIR spectroscopy during biodiesel production in a microreactor. In addition to *on-line* monitoring of the components of the reaction mixture by applying this approach, useful process parameters, such as biodiesel yield or reactant ratio, can be optimized during a continuous production process based on the results obtained in a short time, leading to faster and sustainable process development.

## 2. Materials and Methods

### 2.1. Materials

#### 2.1.1. Chemicals

Edible sunflower oil (Zvijezda, Zagreb, Croatia) was bought in a local supermarket. The FAME mix GLC-10 and a commercial lipase from *Thermomyces lanuginosus* (Lipolase 100 L) were purchased from Sigma-Aldrich Handels GmbH (Vienna, Austria). Dipotassium hydrogen phosphate (K_2_HPO_4_) was purchased from Merck (Darmstadt, Germany). Potassium dihydrogen phosphate (KH_2_PO_4_) was purchased from Lach:ner (Prague, Czech Republic). Glycerol was purchased from Kemika (Zagreb, Croatia), and methanol was purchased from Gram mol d.o.o. (Zagreb, Croatia). SDS (Sodium dodecyl sulphate) was purchased from VWR International (Leuven, Belgium). With the exception of the sunflower oil, chemicals were of analytical grade and were used without any further purification.

#### 2.1.2. Batch Reactor and Microreactors

**Figure 1:**
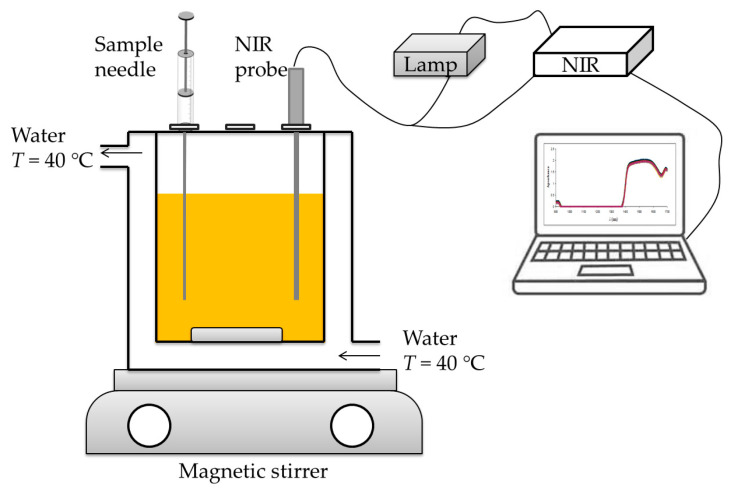
Experimental set-up used for biodiesel production in a batch reactor.

Batch experiments were performed in a glass batch reactor (*V* = 1.5 L), with double glass walls for continuous heating. As shown in Figure 1, the batch reactor was placed on a magnetic stirrer (Witeg MSH 20a, Wertheim, Germany) to ensure constant mixing. The batch reactor has inlets on the top which were used for sampling and connecting the probe to the NIR spectrophotometer.

**Figure 2:**
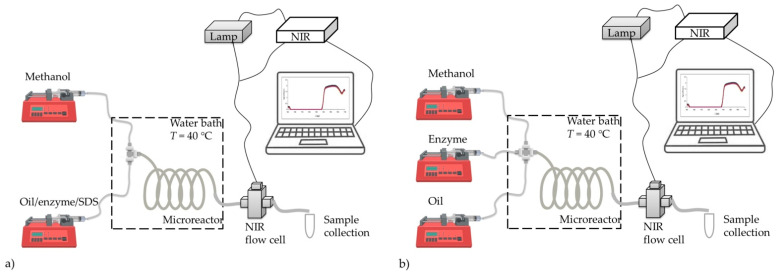
Experimental set-up used for biodiesel production in a microreactor by (**a**) 2-inlet strategy and (**b**) 3-inlet strategy.

**Figure 3:**
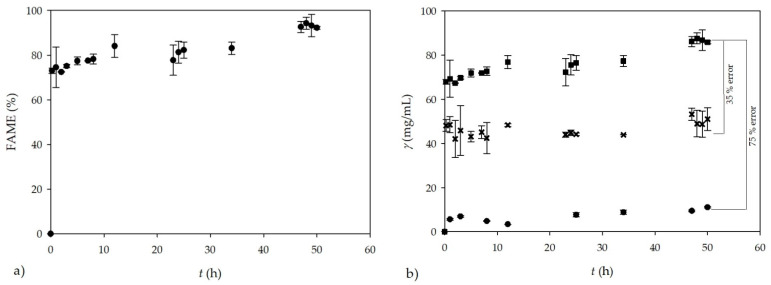
Biodiesel synthesis in a batch reactor (**a**) FAME content and (**b**) comparison of three methods for glycerol determination in collected samples (■—calculated, theoretical glycerol, x—GC, ●—UV-VIS spectrophotometry-based method).

Two microsystems were used for the microreactor experiments. As can be seen in Figure 2, 2-inlet (T-shaped connection, Figure 2a) and 3-inlet (+-shaped connection, Figure 2b) set-ups were used. The PTFE (poly(tetrafluoroethylene)) tube with a constant internal volume of 235.62 μL served as the microreactor. In both microreactor configurations, the inlet tubes containing the substrates/enzyme were connected to the microreactor. Syringes that delivered the fluid into the PTFE tubes were placed on piston pumps (PHD 4400 Syringe Pump Series, Harvard Apparatus, Holliston, MA, USA). The microsystem was placed in a water bath at a temperature of 40 °C.

### 2.2. Methods

#### 2.2.1. Emulsion Preparation

The emulsion was prepared by mixing oil and an enzyme dissolved in a buffer in a volume ratio of 10:1. The oil-in-water emulsion, described in detail in the work by Sharma et al. [30], was prepared using an ultrasonic homogenizer (SONOPULS mini20, Bandelin, Berlin, Germany). The emulsion was prepared by sonicating the mixture for 15 min, at an amplitude of 50% and an operating frequency of 25 kHz. Sodium dodecyl sulphate (SDS) was used as an emulsifier, at a concentration of 0.3 g/L. The homogeneous mixture was acquired.

#### 2.2.2. Measurement of Lipase Activity

Lipase activity was determined spectrophotometrically (UV-1800, Shimadzu, Kyoto, Japan) by measuring the absorbance change at the wavelength of λ = 400 nm [31]. The total determination time was 20 s and the measurements were performed in triplicate. The sample was prepared by adding 100 mL of sample to 3900 mL of 0.05 mol/L Tris-HCl buffer. The measurement was started by adding 50 mL of 0.0375 mol/L 4-nitrophenyl acetate to the homogenized sample–buffer mixture. The lipase activity was calculated taking into consideration the change in the absorbance, the total volume of reaction mixture, the enzyme volume, and a molar extinction coefficient of 0.29866 L/(mmol cm). One unit (U) was defined as the amount of lipase that degrades 1 mmol of 4-nitrophenyl acetate in 1 min.

#### 2.2.3. Initial Experiments—Biodiesel Production in a Batch Reactor and in a Microreactor

Biodiesel is produced by transesterification of a vegetable oil or animal fat with an alcohol, such as methanol or ethanol. In addition to biodiesel, the second product formed in this reaction is glycerol. The set-up for biodiesel production in a batch reactor (*V* = 250 mL, Figure 1), with edible sunflower oil and methanol using Lipolase 100 L as catalyst, was previously described by Gojun et al. [4]. In order to ensure a sufficient amount of methanol in the one-step reaction, the molar ratio of oil to methanol was 1:3.4 (initial concentration of substrates and catalyst: *γ*_methanol_ = 30.60 mg/mL, *γ*_fatty acids_ = 946.52 mg/mL, *γ*_E_ = 0.3 mg/mL). The experiment was performed for two days (50 h) with constant stirring (600 rpm, Witeg MSH 20a, Wertheim, Germany) to ensure biodiesel production. The specific lipase activity (dissolved in a 0.1 mol/L phosphate buffer pH 7.4), was *S.A. =* 1363.45 ± 4.12 U/mg (*corresponding concentration γ*_E_ = 0.3 mg/mL). The concentration was determined spectrophotometrically by measuring the absorbance change at the wavelength of λ = 400 nm (UV- 1800, Shimadzu, Kyoto, Japan). The enzyme activity was tested several times during the experiments, without significant difference during the course of the reaction. A sample volume of 0.5 mL was taken in selected time intervals and analyzed by gas chromatography, UV-VIS spectroscopy, and *off-line* NIR analysis.

The setup for biodiesel production in a microreactor (Figure 2), in which the same substrate and catalyst–lipase as in the batch experiment were used, was previously described by Gojun et al. [4]. Briefly, the first experimental setup consisted of a PTFE coil microreactor with three inlets (+-shape; length:internal diameter = 1.2 m: 0.5 mm with an internal volume of 235.6 µL, Figure 2b). Both substrates, methanol (*γ*_methanol_ = 30.60 mg/mL) in one syringe, oil (*γ*_fatty acids_ = 946.52 mg/mL) in the second syringe, and the catalyst (*γ*_E_ = 0.3 mg/mL) in the third syringe, were introduced into the microreactor as separate streams. When switched to the 2-inlet strategy (Figure 2a), the input of the catalyst was combined with the oil input, by forming an emulsion (as described in Section 2.2.1). The second stream in the 2-inlet strategy was pure methanol. All reaction components were fed into a microreactor separately using high pressure piston pumps (PHD 4400 Syringe PumpSeries, Harvard Apparatus, Holliston, MA, USA). The total flow rates (*q_V_* = 2–235.6 μL/min) of the components in the inlet stream were determined to ensure the same conditions in terms of the initial composition of the reaction mixture as in the experiment performed in a batch reactor (the molar ratio of oil to methanol 1:3.4). The enzyme concentration in the inlet stream was *γ*_E,0_ = 0.3 mg/mL.

#### 2.2.4. Measurements of Fatty Acid Methyl Esters (FAME) and Glycerol Concentrations by Gas Chromatography

FAME and glycerol concentrations were determined according to the method described by Gojun et al. [4] on a gas chromatograph (Shimadzu GC-2014, Tokyo, Japan) equipped with FID and the Zebron ZB-wax GC capillary column (length 30 m, I.D. 0.53 mm, and film thickness 1.00 µm, Phenomenex, Torrance, CA, USA). The carrier gas in this method was nitrogen, at the rate of 1.97 mL/min. With the total determination time of 15 min, the measurement started at the temperature of 180 °C for 1 min, with the column heating up to 230 °C, at the rate of 5 °C min^−1^. The FAME mix GLC-10 was used as the standard for identifying peaks for corresponding FAME. The retention times of FAME compounds are as follows: 7.74 min for palmitic, 10.59 min for stearic, 10.87 min for oleic, 11.58 min for linoleic, and 12.62 min for *α*-linoleic (linolenic). The retention time for glycerol was 9.02 min while applying the same method. To confirm repeatability, all measurements were performed in triplicate. On a 95% confidence interval the results showed no significant difference.

#### 2.2.5. Measurements of the Glycerol Concentration by UV-VIS Spectroscopy

The concentration of glycerol in the produced biodiesel was determined in a cuvette (*d* = 1 cm) using the UV-VIS spectrophotometer Shimadzu UV-1601 (Tokyo, Japan) at the wavelength *λ* = 410 nm, according to the method described by Bondioli and Della Bella [32]. To determine repeatability, all measurements were performed in triplicate. At a confidence interval of 95% the results showed no significant difference.

#### 2.2.6. Measurements of the Glycerol Concentration by NIR Spectroscopy

The glycerol concentration was measured with the NIR-128L-1.7-USB/6.25/50 μm spectrophotometer (Control Development, South Bend, IN, USA) with installed Control Development software Spec32, using a halogen light source (HL-2000) and 2 mm path length. The integration time was set to 0.03 s with sample average set to 10. For *off-line* determination by NIR spectroscopy, the sample was taken at the end of the biodiesel production process, placed in a quartz cuvette (*d* = 1 cm), and placed in a holder with a black metal lid to avoid interference from external light. During the batch experiment and *on-line* screening of the NIR spectrum, the NIR probe was immersed directly in the reaction mixture. The probe was connected to the NIR spectrophotometer. In the microreactor experiment, the 100 μL quartz flow cell of the NIR spectroscope (Hellma Analytics, Müllheim, Germany), with a spectral range from 200 nm to 2500 nm, was connected to the outlet stream of the microreactor. The samples were not treated (mechanically or chemically) prior to analysis. All sample spectra were recorded in the wavelength range from *λ* = 904 nm to *λ* = 1699 nm, with a step size of 1 nm, and each spectrum was correlated to the sample with a different content of glycerol. To determine repeatability, all measurements were performed in triplicate, meaning that three consecutive spectra were recorded for each sample.

#### 2.2.7. Statistical Analysis and Modeling

##### Principal Component Analysis (PCA)

PCA, which is commonly used to distinguish similarities and differences between samples, was applied in this study to reduce the number of variables that were later used for ANN modeling. The idea behind the PCA was to transform significant data from the spectra and express them as so-called principal components or factors (PC factors)—a set of new orthogonal variables. In order to capture as high a percentage of the variations as possible [33], meaning that none of the vital information is lost, the first 10 factors, which were responsible for 99% of the variations, were later used for ANN modeling. The raw NIR spectra were used to perform the PCA by the Statistica v.14.0 software (StatSoft, Tulsa, OK, USA). The PCA was performed for different individual sets of experiments.

##### Artificial Neural Network (ANN) Modeling

The PCA factors were next used as inputs to ANNs, to monitor glycerol formation during biodiesel synthesis. Multilayer perceptron networks were developed in Statistica v.14.0 software (StatSoft). Based on the PCA, the first 10 PCA factors, which were responsible for 99% of the variance, were selected and used as input variables with the extraction condition data. The ANN training was performed with the separation of data into training, test, and validation sets at the 60:20:20 ratio with random data selection done by software. The back error propagation algorithm available in Statistica v.14.0 was applied for model training. The model performance was evaluated based on *R*^2^ and root mean squared error (RMSE) values for training, test, and validation.

#### 2.2.8. Biodiesel Production in a Batch and in a Microreactor Followed by *Off-Line* Determination of Glycerol Concentration

The set-up for biodiesel production in a batch reactor was the same as the one described in Section 2.1.2 (Figure 1). Samples were collected in regular time intervals, put on ice to stop the reaction, and part of the sample was analyzed by NIR spectroscopy. Then, the rest of the sample was prepared for GC analysis.

The set-up for biodiesel production in a microreactor was the same as the one described in Section 2.1.2 (Figure 2). Samples from the microreactor outlet were collected in vials, cooled on ice to stop the reaction, and part of the sample was analyzed by NIR spectroscopy. After that, the rest of the sample was prepared for GC analysis.

#### 2.2.9. Biodiesel Production in a Batch and in a Microreactor Followed by *On-Line* Determination of Glycerol Concentration

The set-up for biodiesel production in a batch reactor was the same as the one described in Section 2.1.2 (Figure 1). For the NIR *on-line* analysis, the NIR probe was immersed into the reaction mixture for the entire duration of the process (*t* = 50 h). Additionally, samples were collected in regular time intervals for the GC analysis, which was carried out subsequently.

The set-up for biodiesel production in a microreactor was the same as described in Section 2.1.2 (Figure 2a), except that the output stream was connected to the 100 μL NIR flow cell (Hellma Analytics, Müllheim, Germany) and the reaction medium was scanned *on-line* with NIR spectroscopy. The output of the flow cell was connected to the vial where samples were collected on ice for the *off-line* measurements by GC and NIR spectroscopy.

Additionally, the experimental set-up was performed with two inlets (T-shape, length: internal diameter = 1.2 m: 0.5 mm with an internal volume of 235.6 µL), so that the excess of methanol in the system could be changed, the same as in previous research conducted by Gojun et al. [4]. Two oil to methanol molar ratios were investigated with this experimental set-up, the excess of methanol mentioned earlier (oil:methanol 1:90) and the stoichiometric oil to methanol molar ratio (oil:methanol 1:3.4). An emulsion of oil and enzyme dissolved in a 0.1 mol/L potassium-potassium phosphate buffer (pH 7.4) was placed in one syringe and methanol was placed in the second syringe. The emulsion was prepared by mixing oil and the enzyme dissolved in the buffer, at the volume ratio of 10:1. The concentration of SDS in the emulsion was 0.1 g/L and the emulsion was prepared on a laboratory shaker (Tehtnica, Vibromix 313EVT, Železnik, Slovenia) for 25 min at 600 rpm. The output stream of the microreactor was connected to the 100 μL NIR flow cell (Hellma Analytics, Müllheim, Germany) and the reaction medium was scanned with NIR spectroscopy *on-line*. The output of the flow cell was connected to the vial in which samples were collected on ice for the *off-line* measurements by GC and NIR spectroscopy.

#### 2.2.10. Evaluation of *On-Line* Measurement of Glycerol Concentration for Experiments Performed in a Microreactor

To further evaluate the *on-line* measurement of the glycerol concentration, experiments in a +-shape microreactor (Figure 2b) were performed with two different concentrations of oil in the inlet stream and for different residence times, respectively.

In an experiment performed with two different concentrations of oil in the inlet stream, the constant flow rate of 23.56 μL/min (residence time of *τ* = 10 min) was applied. Concentrations of methanol and enzyme in the inlet streams were *γ*_methanol_ = 30.60 mg/mL and *γ*_E_ = 0.3 mg/mL, respectively. In the first part of the experiment, the concentration of oil in the inlet stream was *γ*_fatty acids_ = 946.52 mg/mL. After the steady state (> 4*τ*) was established, i.e., after 48 min of the experiment, the second phase of the experiment started. Namely, the oil concentration in the inlet stream was reduced two-fold and it was *γ*_fatty acids_ = 473.26 mg/mL. The experiment continued for an additional 4*τ* until a new steady state was established. In this experiment scanning by NIR spectroscopy was made every 2–4 min, while samples for GC were collected approximately every 6 min.

In the experiment performed for different residence times, the total flow rate was changed in the range of 5.9–235.62 μL/min (residence times of *τ* = 40–1 min). Concentrations of compounds in inlet streams were *γ*_methanol_ = 30.60 mg/mL, *γ*_E_ = 0.1 mg/mL, and *γ*_fatty acids_ = 946.52 mg/mL, respectively. The flows were changed before the steady state was established. A total of 16 samples were collected for *off-line* analysis on GC, while scanning by NIR spectroscopy was made every 2–4 min. Prior to analysis on the GC, samples were placed on ice to stop the reaction by enzyme inactivation.

## 3. Results and Discussion

### 3.1. Biodiesel Synthesis in a Batch Reactor and Comparison of Analytical Methods

Biodiesel was synthesized in a batch reactor (*V* = 250 mL) using sunflower oil and methanol in excess as substrates and the enzyme lipase as the biocatalyst. During the process, the dynamic change of biodiesel yield (% FAME) was monitored and it is presented in Figure 3a. At the end of the process, successful biodiesel production was performed, resulting in a biodiesel yield of *I* = 93 ± 2.5% after 50 h.

Based on the stoichiometry of the reaction and GC measurements of FAME concentration, the theoretical glycerol concentration in collected samples was calculated. The obtained biodiesel yield at the end of the transesterification performed in a batch reactor (Figure 3a) corresponds to the FAME concentration of *γ*_FAME_ = 1041.99 ± 1.18 mg/mL, which is consistent with the calculated (theoretical) total glycerol concentration of 93 mg/mL. The obtained results are in accordance with the usual biodiesel production by transesterification, which produce, on average, 1 kg of glycerol per 10 kg of biodiesel. To confirm the theoretical glycerol concentrations calculated based on the GC measurements from FAME, two additional analytical methods were used for *off-line* measurement of total glycerol concentration. Usually, the concentration of glycerol produced during biodiesel synthesis by transesterification is monitored by GC and/or UV-VIS spectrophotometric methods, respectively [9,10,16].

Glycerol concentrations obtained by GC and UV-VIS spectrophotometric measurements are shown in Figure 3b and compared to the calculated theoretical glycerol concentrations. As can be seen from Figure 3b, the theoretical concentration of total glycerol during the transesterification process was in the range of 70–93 mg/mL. The measurements performed by GC resulted in glycerol concentrations in the range from 45 to 65 mg/mL, which is 35% less than the calculated theoretical value. The reason for this difference is probably the high rate of glycerol precipitation and sedimentation during the collection and preparation of samples for GC analysis, resulting in a significant loss of glycerol in the analyzed sample. The measurements performed by the UV-VIS spectrophotometric method show an even greater deviation (75%) from the theoretical values, as the preparation of samples requires even more steps, resulting in a greater glycerol loss, and consequently leading to greater errors. Based on the presented figures, both *off-line* analytical methods were proved to be inadequate for quantifying the total glycerol formed during biodiesel production by transesterification. This was also observed by Gelinski et al. [34], who reported that the majority of results during glycerol quantification by the UV-VIS method were below the expected level. The main disadvantage of NIR spectroscopy is that it needs to be validated by another technique, which is the basic requirement for NIR spectroscopy to be used independently for measurements in further experiments. In this research, theoretical concentrations of glycerol were used to validate the results of NIR spectroscopy. On the other hand, NIR spectroscopy does not require additional sample preparation, which is the main advantage of this method. Consequently, NIR spectroscopy is less costly and time consuming compared to other analytical methods [19,25,27].

### 3.2. NIR Spectroscopy for the Measurements of Glycerol Concentration in Model Samples

As an alternative to the previously mentioned method, based on calculated theoretical glycerol concentrations, the measurement of glycerol concentration was performed by NIR spectroscopy. The first step was to record the NIR spectra based on the range of different glycerol concentrations. The set of 20 samples performed in triplicate with different glycerol concentrations (model samples) ranging from 0 to 100 mg/mL was prepared to obtain the calibration model for *on-line* monitoring of biodiesel production by transesterification. Eleven samples performed in triplicate resulting in 33 samples were performed for glycerol concentrations from 0 to 100 mg/mL, and nine samples performed in triplicate resulting in 27 samples were performed for glycerol concentrations from 0 to 0.035 mg/mL.

**Figure 4:**
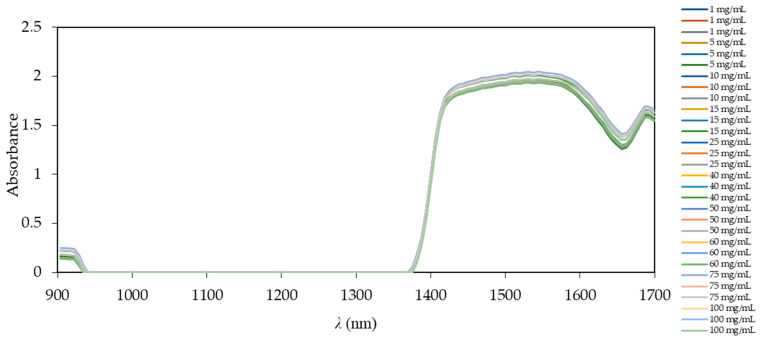
NIR spectra of glycerol concentrations in model samples ranging from 0 to 100 mg/mL. Triplicates of each concentration are given in the legend.

Model samples were prepared from crude glycerol, diluted in ultra-purified water in the range from 0 to 100 mg/mL. This concentration range was chosen in order to cover all expected glycerol concentrations that could occur during the usual course of the biodiesel production process [35]. Before NIR measurements, the calibration to dark and light source was performed. The obtained spectra from NIR spectroscopy without any pre-processing for all tested samples are shown in Figure 4.

Overlapping spectra in Figure 4 show that there is a region where the spectra do not differ (941–1370 nm), as they are simply different glycerol concentrations in ultra-purified water. The main difference in the spectra can be found in the region below 941 nm and in the region from 1370 to 1700 nm. This region corresponds to the stretching and deformation vibrations of –CH, –CO, and –OH groups present in glycerol. It is also noted that the spectra show significant overlapping and high similarity in shape and intensity. In order to find similarities and differences between samples in this part of the spectrum, further analysis of the main components can be performed without pre-processing of the NIR spectra using the PCA method. This method can reduce a great number of variables (from several hundred to tens) and remove noise [36]. Nowadays, pre-processing of NIR spectra is commonly used for biodiesel synthesis monitoring using NIR [34]. Based on our previous experience, the use of pre-processing techniques, such as smoothing, on the first or second derivative can lead to a loss of sometimes vital information [23,24]; thus, in this work, we only used raw NIR spectra for further analysis. Factors obtained from PCA analysis of the NIR spectra were further used as inputs for ANNs, in order to monitor *on-line* glycerol formation during biodiesel synthesis.

To develop ANNs, it was necessary to select the neural network that could process the obtained data. Even though different ratios for training, test, and validation (50:30:20, 60:20:20, 70:15:15, 70:20:10) were tested on available experimental results, the highest *R*^2^ and the lowest root mean square error (RMSE) was obtained for the 60:20:20 ratio, respectively. In all of the above cases, the data were randomly selected by the software from the obtained theoretical values of glycerol. Table 1 shows five obtained ANNs (10-13-1, 10-9-1, 10-6-1, 10-8-1, and 10-7-1) and their characteristics. The first number in the network architecture indicates the input of ANN, which, in this case, is the number of factors obtained by the PCA analysis (10), the next number is the number of neurons in the hidden layer, which was set in the range of 6–13, and the last is the number of outputs (glycerol concentration). Based on the *R*^2^ values for training, test, and validation and their errors, the best network was selected. Even though all the proposed neural networks listed in the table have extremely high values, i.e., all values of training accuracy, testing, and validation are greater than 0.9, neural network number 3 was chosen because of the smallest number of neurons in the hidden layer, which provides additional network stability. Furthermore, in the case of the training neural network number 2, and in the case of testing neural networks number 1 and 4, the higher *R*^2^ values are obtained at validation. On the contrary, the selected neural network number 3 has the highest precision value. Moreover, very importantly, this network is characterized by the smallest error.

As mentioned earlier, the 10-6-1 architecture means that there were 10 factors obtained by PCA for the input variables, six neurons were in the hidden layer, and the glycerol concentration was the only output. In this case, the first 10 factors of the PCA analysis, which explained 99% of the variability in the data, were used as inputs for ANN. In this case, ANN that used 60% of the total data for learning, 20% for testing, and 20% for validation, and have 6–13 neurons in the hidden layer, were found to be the most suitable.

**Figure 5:**
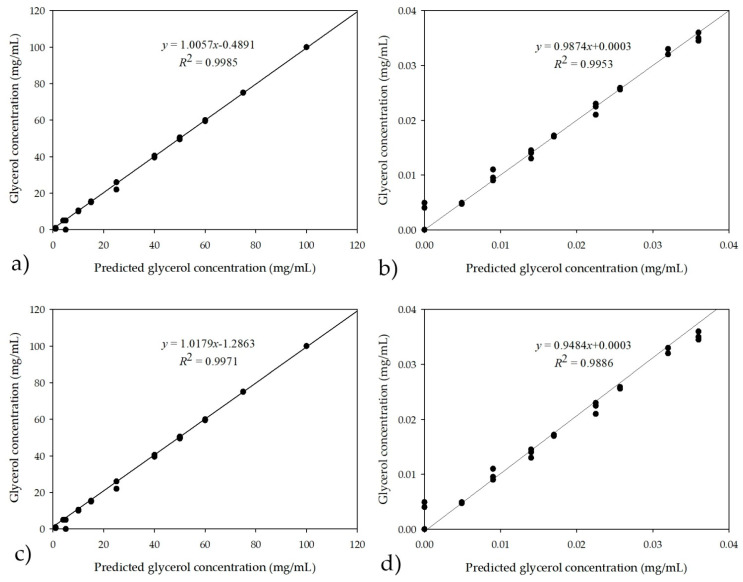
Comparison of glycerol concentration with prediction of selected ANN for different concentration ranges (**a**) from 0 to 100 mg/mL and (**b**) from 0 to 0.035 mg/mL and subsampling ANN prediction for different concentration ranges (**c**) from 0 to 100 mg/mL and (**d**) from 0 to 0.035 mg/mL.

Figure 5a shows the correlation between the prediction of the glycerol concentration using the selected neural network and the experimental values for glycerol concentrations in the model samples ranging from 0 to 100 mg/mL.

**Figure 6:**
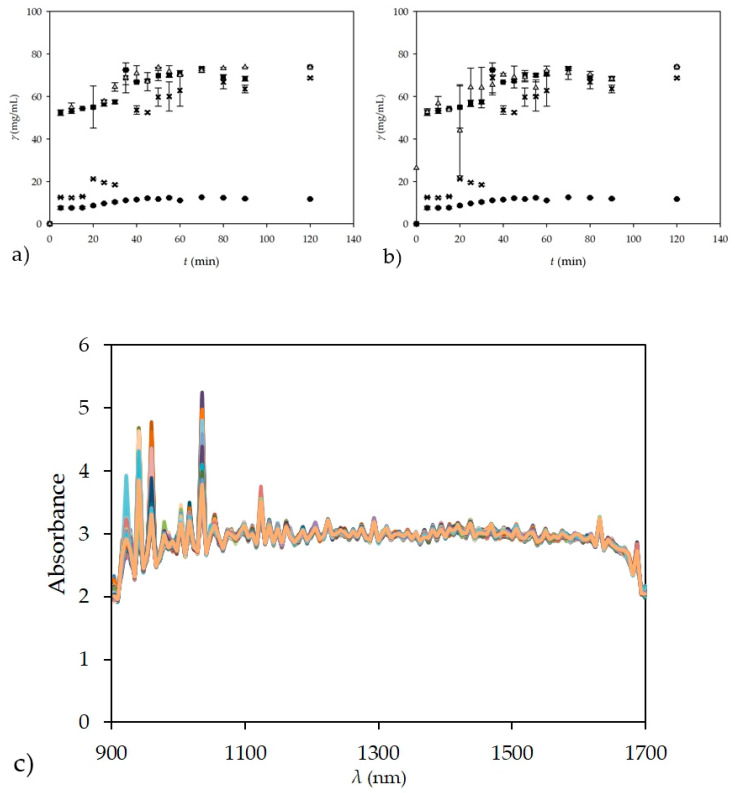
(**a**) Comparison of glycerol concentrations obtained by different methods during the biodiesel production process performed in a batch reactor; (**b**) comparison of glycerol concentrations obtained by different methods during the biodiesel production process performed in a batch reactor using subsampling ANN for NIR spectroscopy (■—calculated, theoretical glycerol, x—gas chromatography, ●—UV-VIS spectrophotometry, ▲—NIR spectroscopy); and (**c**) NIR spectra of the biodiesel production process during a batch experiment.

To verify that the lower calculated theoretical glycerol concentrations could be determined using an NIR spectrometer, dilutions of the model samples were made and the glycerol concentrations in the samples were in the range of 0–0.03 mg/mL (Figure 5b). As in the previous case, all concentrations were performed in triplicate and the PCA was performed first from the recorded NIR spectrum data, followed by ANN. The neural networks that had a total data ratio of 60:20:20 randomly selected by the software for training, testing, and validation also performed the best, as was the case of the higher range of glycerol concentrations (0–100 mg/mL). In this case, the neural network which was selected had *R*^2^ values of 0.9996, 0.9974, and 0.9937 for learning, testing, and validation, with errors of 0.0000, 0.0002, and 0.0030, respectively. Because the software randomly selects data for training, testing, and validation based on user-defined ratios, all three samples (triplicates) recorded in a single interval could be used for validation. The main question remains whether this ANN is valid. From Figure 6a, it is easy to see whether the values of ANN show over-fitting or under-fitting, leading to higher or lower results for the calculated theoretical glycerol. Of course, one must look at the *R^2^* values and error values to determine if ANN is overtrained or undertrained. When ANN is overtrained, situations such as high *R^2^* values for training with lowest errors occur, while *R^2^* values for testing and validation are very low and have high errors. When ANN is undertrained, low *R^2^* values for training occur with high error values, while high *R^2^* values for testing or validation are observed with low error values. To avoid selecting triplicate samples from the same time interval by random software generation, one must perform the predictive tests of the ANN model in the Statistica software, with the predictive datasheet showing which samples were used for training, testing, and validation. The other way to avoid this is to use some kind of cross-validation, such as subsampling strategy, for building predictive models. For this purpose, subsampling was performed separately for both ANN (for higher and lower glycerol concentrations) using six neurons in the hidden layer, with the number of subsamples set at five and the subsample in the ratio 60:20:20 using the random subsampling method and the seed for subsampling 1000. In all cases (in this and following cases), the dataset used was exactly the same as for ANNs without subsampling, i.e., the PCA results from the recorded NIR spectra. The results of the five ANNs for the higher glycerol concentrations in terms of *R^2^* values ranged from 0.9999 to 0.9992 for training, 0.9995 to 0.9926 for testing, and 0.9956 to 0.7500 for validation, with the best results obtained for ANN number 4, which had *R^2^* values 0.9999, 0.9995, and 0.9956 with errors of 0.0000, 0.0013, and 0.0056 for training, testing, and validation, respectively. For the lower glycerol concentration, five ANNs were identified whose *R^2^* values ranged from 1.0000 to 0.9526 for training, 0.9982 to 0.8764 for testing, and 0.9968 to 0.2057 for validation, with the best results obtained for ANN, which had *R^2^* values of 0.9984, 0.9957, and 0.9935 with errors of 0.0000, 0.0000, and 0.0000 for training, testing, and validation, respectively. The results of the subsampling strategy for predicting higher and lower glycerol concentrations are shown in Figure 5c,d, respectively.

### 3.3. NIR Spectroscopy for Glycerol Off-Line Monitoring of Real Samples for Experiments Performed in a Batch Reactor

After obtaining a good agreement between the results measured by NIR spectroscopy and the data obtained by prediction of the selected ANN for known glycerol concentrations in model samples, NIR spectroscopy was applied for *off-line* analysis of real samples from the biodiesel production process. Biodiesel production was again performed in a batch reactor and the samples were taken out of the reactor at different time intervals. The glycerol concentration was measured in collected samples by GC, UV-VIS spectrophotometry, and NIR spectroscopy by previously described methods. Furthermore, the concentration of FAME was measured in all samples by GC and used to calculate the theoretical glycerol concentration. Triplicate samples were taken at 5 min intervals during the first 60 min and then at 10 min intervals until 90 min with the last samples taken at 120 min, resulting in 51 samples (17 different times × 3 samples per time). Glycerol concentrations obtained by different methods are shown in Figure 6a, and the spectra obtained by NIR spectroscopy without pre-processing for all tested samples during the batch experiment are shown in Figure 6b.

Glycerol concentrations measured by the UV-VIS spectrophotometer again showed the largest deviation from the theoretically calculated glycerol concentration. For measurements performed by GC, the deviations in the glycerol concentration were slightly smaller but still present, probably due to its precipitation and sedimentation during sample preparation and analysis. On the other hand, the glycerol concentrations obtained by NIR spectroscopy coupled with ANN predictions that were performed using theoretical glycerol concentration are practically equal with the calculated theoretical glycerol concentrations; thus, demonstrating the accuracy of the NIR method. For this experiment an ANN model based on calculated theoretical values was performed with the best prediction of experimental values for ANN that had architecture 10-9-1. As in the previous case, the data ratio was 60:20:20, with data randomly selected by software, for training, testing, and validation. *R*^2^ values of 0.9733, 0.9482, and 0.9412 were obtained for training, testing, and validation with error values of 0.0059, 0.0203, and 0.0098, respectively. The main reason for the accuracy of the NIR method, among other reasons, is probably the fact that the glycerol concentration was measured directly in the sample, without the need to prepare the sample before the analysis itself.

As shown in Figure 1, an NIR probe was inserted into the reactor for the batch process. From the comparison of Figure 4 and Figure 6c, it is clear that the spectra of glycerol in ultra-purified water are significantly different from the spectra obtained in batch measurements. The reason for this is the much more complex system in the batch reactor due to all the components present, such as the edible sunflower oil, methanol, buffer, enzyme, FAME, and glycerol. The replacement of ultra-purified water with vegetable oil adds additional peaks, as does the addition of the enzyme and any other component, resulting in such a difference not only in the 941–1370 nm range, which showed no difference with ultra-purified water, but also in the intensity of the NIR spectra. Some might argue that the spectra might also contain some kind of noise effect, and the best way to deal with noise is to first smooth the spectra to avoid this. However, the goal of this work was to get the best out of the unmodified spectra, i.e., with any additional pre-processing, and thus, test not only the ability of the NIR instrument, but also the ability of the ANN to work with such complex data. In addition, noise could easily affect the ANN, since learning from data with noise sometimes yields high *R^2^* values for training, but the final result in validation is not as high as the results obtained in this experiment. As in the previous case with different concentration ranges of glycerol concentrations, subsampling of the data was performed for ANN, which, in this case, had nine neurons in the hidden layer, with the number of subsamples set to five, and the size of the subsamples in the ratio 60:20:20 using the random subsampling method and the seed for subsampling 1000. The most suitable network was network number 3, with *R*^2^ values of 0.9785, 0.8968, and 0.8348 with errors having values of 0.0063, 0.0074, and 0.2511 for training, testing, and validation, respectively. These results are shown in Figure 6b. In this case, it is clear that network selection without cross-validation can lead to better results, as one can select the one with the highest *R^2^* values from hundreds of different ANNs. This can sometimes be misleading, especially in cases with smaller datasets. In these cases, subsampling can provide much more reliable results; although they may not be as successful in terms of *R^2^* values, they ensure the right kind of ANN validation.

### 3.4. On-Line Measurements of the Glycerol Concentration by NIR Spectroscopy in a Batch Reactor and in a Microreactor

Considering the successfully performed *off-line* measurements of the glycerol concentration by NIR spectroscopy, *on-line* measurements of glycerol concentrations in a batch reactor and in a microreactor were performed during the course of the transesterification process. During the batch experiment, the probe was immersed in the reactor throughout the process, and spectra were recorded every five minutes for the first 60 min, followed by 10 min recording intervals up to 90 min, and the last spectra was recorded after 120 min resulting in 51 samples (17 different times × 3 samples per time). In order to prevent outside light interference, the reactor was enveloped with aluminum foil. For the experiment performed in a microreactor, the output stream was connected to the covered flow cell of the NIR spectroscope; thus, preventing any interference of outside light source. As a result, the reaction medium in the outlet stream of the microreactor could be analyzed *on-line* with NIR spectroscopy.

**Figure 7:**
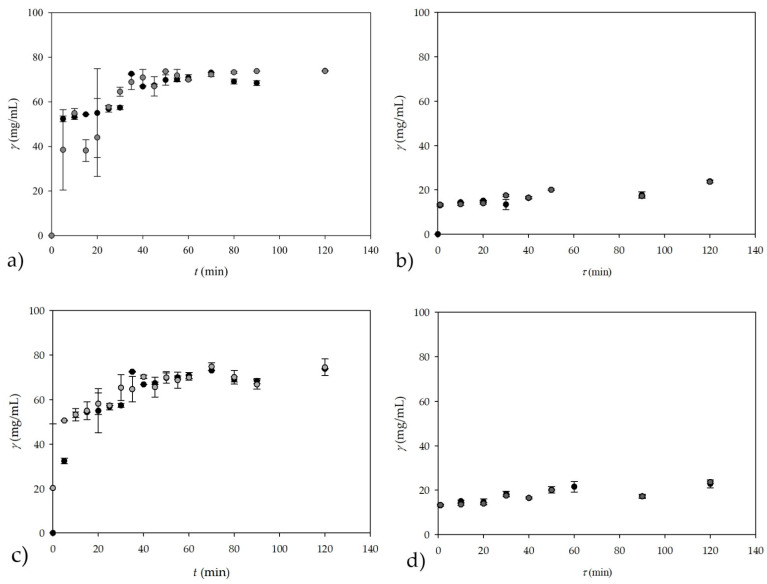
*On-line* measurement of glycerol concentration by NIR spectroscopy during the transesterification reaction (oil to methanol molar ratio = 1:3.4) carried out (**a**) in a batch reactor; (**b**) in +-shaped microreactor; (**c**) in a batch reactor using subsampling ANN for NIR spectroscopy; and (**d**) in +-shaped microreactor using subsampling ANN for NIR spectroscopy (●—calculated, theoretical glycerol, ●—NIR spectroscopy).

A comparison of the calculated theoretical values of the glycerol concentration and the glycerol concentration measured *on-line* by NIR spectroscopy are shown in Figure 7a for the experiment conducted in a batch reactor.

Obviously, a good agreement between the glycerol concentrations measured by *on-line* NIR spectroscopy and the calculated theoretical values was obtained.

Considering the successfully performed *on-line* measurements in a batch reactor, the possibility of *on-line* measurements of the glycerol concentration in the continuous reaction system, i.e., in the PTFE coil microreactor with three inlets, was also investigated. The corresponding results are shown in Figure 7b. The experiment in the +-shaped microreactor was performed for eight different residence times, resulting in 24 samples (triplicate samples for each residence time), and the spectra of the samples showed the same trend as those shown in Figure 6b for the batch reactor. To obtain a larger dataset, the data from the batch reactor (17 samples in triplicate, resulting in 51 samples) and the data from the +-shaped microreactor (24 samples) were combined, resulting in a matrix of 75 samples x 795 wavelengths, which was later used for ANN modeling, after PCA was performed. The reason for coupling data from different experiments was to test if it is possible to monitor glycerol concentration in different systems. As in the experiment conducted in a batch reactor, the *on-line* measurement of the glycerol concentration by NIR spectroscopy shows very good agreement with the theoretically calculated values of the glycerol concentration.

The best ANN model that was used for both experiments was obtained once again with ANN that had a 60:20:20 ratio of randomly selected data by software for training, testing, and validation with an ANN structure of 10-6-1. *R^2^* values of 0.9886, 0.9735, and 0.9375 with errors of 0.0013, 0.0036, and 0.0111 were obtained for training, testing, and validation, respectively. In addition, ANN model 4, obtained from performing ANNs with five subsamples and the subsample size in the ratio of 60:20:20 with six neurons in the hidden layer, also provided very promising results with *R^2^* values of 0.9868, 0.9588, and 0.9026 for training, testing, and validation, respectively, with corresponding errors of 0.0031, 0.0042, and 0.1628. The results of the subsampling ANN model are shown in Figure 7c for the batch reactor and in Figure 7d for the +-shaped microreactor. This is further evidence that NIR spectroscopy is a good method for measuring glycerol concentration in a batch reactor and in a continuous microreactor by means of *off-line* and *on-line* measurements.

**Figure 8:**
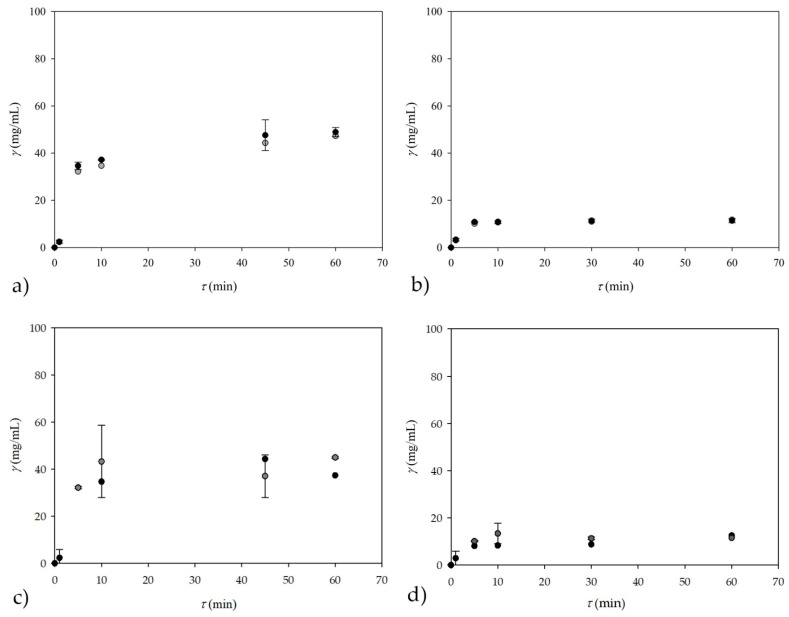
*On-line* measurement of glycerol concentration by NIR spectroscopy during the transesterification reaction carried out in a T-shaped microreactor. (**a**) Experiment with the high surplus of methanol (oil to methanol molar ratio = 1:93); (**b**) experiment with a stoichiometric oil to methanol ratio (oil to methanol molar ratio = 1:3.4); (**c**) experiment with the high surplus of methanol (oil to methanol molar ratio = 1:93) using subsampling ANN; and (**d**) experiment with a stoichiometric oil to methanol ratio (oil to methanol molar ratio = 1:3.4) using subsampling ANN (●—calculated, theoretical glycerol, ●—NIR spectroscopy on-line measurement).

Based on the good results obtained with NIR, on the basis of *on-line* measurements of the glycerol concentration for experiments performed in a PTFE coil microreactor with three inlets, an additional two experiments were conducted in a different microreactor configuration, namely, in a microreactor with two inlets. In this experiment, the first inlet stream consisted of methanol and the second inlet stream consisted of the emulsion formed from oil and an enzyme dissolved in a buffer with the addition of an emulsifier (SDS). In one of our previous works [4], we demonstrated that if a large excess of methanol was used (oil to methanol molar ratio 1:90) the reaction can be shifted towards product formation. Consequently, higher yields will be achieved in comparison to processes with the stoichiometric ratio of substrates for the same residence time. Such methanol excess is not possible in a batch process because the enzyme is significantly inhibited by higher methanol concentrations. Namely, lipase-catalysed biodiesel production is only possible in systems where the lipase is somehow protected from the high methanol content. This could be realized in a microreactor equipped with three inlets in which enzyme, methanol, and oil are fed as separate streams, or in a microreactor equipped with two inlets in which the mixture of the enzyme and oil is fed as one stream while methanol is fed separately. In those microreactor systems, the enzyme was not in direct contact with the methanol and the reaction occurred only at the surface of phases. Having in mind that the *on-line* measurement of glycerol concentration by NIR spectroscopy during the transesterification reaction in a +-shaped microreactor is successfully performed (Figure 7b), additional experiments were performed in a T-shaped microreactor to show the applicability of NIR spectroscopy for measurements of the glycerol concentration in a different microreactor configuration. Additionally, different oil to methanol ratios were also tested in order to investigate the influence of different concentration profiles on the reliability of NIR spectroscopy (Figure 8a,b). In the transesterification reaction performed in a T-shaped microreactor, NIR spectroscopy could also be influenced by the presence of the emulsifier needed for the preparation of a stable emulsion of oil and lipase (one of the two inlet streams).

For this, *on-line* NIR spectroscopy measurements coupled with an ANN (with the architecture of 10-13-2 and the 60:20:20 ratio for training, testing, and validation) prediction model were compared with theoretical glycerol concentrations for the experiment performed in a microreactor with two inlets (Figure 8a,b). For this experiment, theoretical glycerol values from previous microreactor experiment (Figure 7b) coupled with new data for theoretical glycerol values obtained in two experiments with a T-shaped microreactor were used for later modeling. To perform the ANN analysis, a data matrix was first created consisting of 27 samples from Figure 7b, 18 samples from Figure 8a, and 18 samples from Figure 8b, followed by PCA prior to ANN modeling. As in the previous cases, the first 10 factors from PCA were used as input variables for the ANN, resulting in a data matrix of 63 samples and 10 factors with, in this case, two output variables (glycerol concentration and residence time) to simultaneously test whether it was possible to predict not only glycerol concentration but also residence time.

It can be seen from the ANN architecture that there were two outputs in this case, the first one being glycerol concentration, and the other being residence time. Results for this ANN in terms of *R*^2^ values were 0.9999, 0.9746, and 0.9408 with errors of 0.0000, 0.0053, and 0.1531, for training, testing, and validation, respectively, which was combined for glycerol concentration and residence time.

Since this ANN is a combination of two outputs, for the first output (glycerol concentration), *R*^2^ values for training, testing, and validation were 0.9999, 0.9495, and 0.9463, while for the residence time, *R*^2^ values for training, testing, and validation were 0.9999, 0.9996, and 09354.

In the case of the subsampling strategy (Figure 8c,d), which was performed with five subsamples and a subsample size ratio of 60:20:20 with 13 neurons in the hidden layer, the results were lower in terms of *R*^2^ values for training, testing, and validation, resulting in values of 0.9997, 0.9836, and 0.9051 with errors of 0.0002, 0.0089, and 0.1202, respectively. Although the difference is not that great in term of values for validation in both cases, noticeable difference can be seen when looking at the results for the first output (glycerol concentration), where *R*^2^ values of 0.9999, 0.9713, and 0.8146 were obtained for training, testing, and validation; thus, presenting a problem, which is most probably caused from working with smaller datasets. The results for time were, in the second case, much better with the *R*^2^ values of 0.9996, 0.9959, and 0.9956 for training, testing, and validation.

Although the theoretical glycerol concentrations in the second case (subsampling strategy) is a bit lower than expected, with additional data from the future experiments, glycerol concentration and residence times correlated with *on-line* NIR measurements could have potential applicability of ANN model simulations for *on-line* estimations of glycerol concentrations and/or residence time during continuous biodiesel production in a microreactor by means of transesterification. These results show that *on-line* NIR measurements are applicable in systems with different configurations and for experiments performed at different concentrations of reactants and other components of the reaction mixture, without the need for any adjustments.

### 3.5. On-Line Measurement of Glycerol Concentrations—Prediction by ANN Based on NIR Spectroscopy

**Figure 9:**
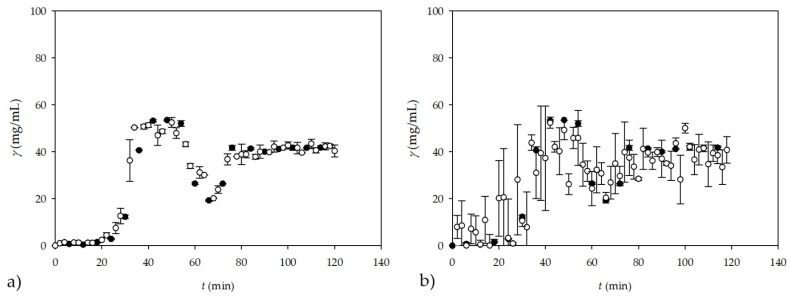
*On-line* measurement of glycerol concentration performed as (**a**) prediction by ANN based on NIR spectroscopy for experiments carried out in a +-shape microreactor for two different concentrations of oil in inlet stream and (**b**) prediction by subsampling ANN based on NIR spectroscopy for experiments carried out in a +-shape microreactor for two different concentrations of oil in inlet stream (●—theoretical glycerol calculated from experimental data ○—ANN predictions).

In order to further investigate NIR spectroscopy potential, an additional experiment was performed in a microreactor for two different concentrations of oil in the inlet stream (described in Section 2.2.9). For this purpose, the ANN model was developed based only on the calculated theoretical values of glycerol and the recorded NIR spectra for those samples were developed. Although the NIR spectra were recorded every 2 to 4 min, each sample was recorded in triplicate because each sample can be recorded within 10 s, allowing more accurate predictions. In this case, 53 samples were recorded in triplicate, resulting in a total of 159 samples. On the GC, glycerol concentration was measured in 60 samples, leaving 99 samples that were recorded by NIR spectroscopy only. As in the previous cases, PCA was first performed for all 159 samples followed by ANN. Furthermore, the first 10 factors obtained by PCA were used as inputs, while the theoretical glycerol concentration was used as the output variable for ANN. This was only the case for known glycerol concentrations obtained by GC measurements, while all other concentrations were predicted by ANN. The software randomly divided the data into training, testing, and validation in a 60:20:20 ratio. Obtained ANN consisted of 10 input layers (the first 10 PCA factors that were derived from theoretical values of glycerol), 13 neurons in the hidden layer, and an output layer, representing the glycerol concentration, with *R*^2^ values of 0.9781, 0.9761, and 0.9530 and errors of 0.0042, 0.0097, and 0.0160 for training, testing, and validation, respectively. This ANN was used to predict theoretical glycerol concentrations recorded only by NIR spectroscopy, meaning that the predicted values were not measured by GC or any other method. The results of the ANN predictions of glycerol concentrations are shown in Figure 9a. The results of the subsampling strategy, in which the number of subsamples was set to five and the subsample was set to a 60:20:20 ratio with 13 neurons in the hidden layer, are shown in Figure 9b. In this case *R*^2^ values were 0.9853, 0.9437, and 0.8811 for training, testing, and validation with corresponding errors of 0.0045, 0.0179, and 0.0700. When looking at both figures, it is clear that for this kind of prediction much more data will be needed for the training and testing of ANNs in order to make suitable validations and predictions.

It can be observed that the ANN predictions based on the NIR spectra are in accordance with the experimental data and the highest values are observed when both steady states are achieved around 48 and 100 min, respectively. The ANN predictions describe very well the change in glycerol concentration that happened when the oil concentration in the inlet stream was reduced. In addition to the dynamic change, the values obtained by the ANN predictions also describe the steady state part of the process (Figure 9). Moreover, regardless of the different dynamics of sampling (for *off-line* measurements) and recording the NIR spectrum (for *on-line* measurements), there is a clear correspondence between the results of *on-line* and *off-line* measurements.

**Figure 10:**
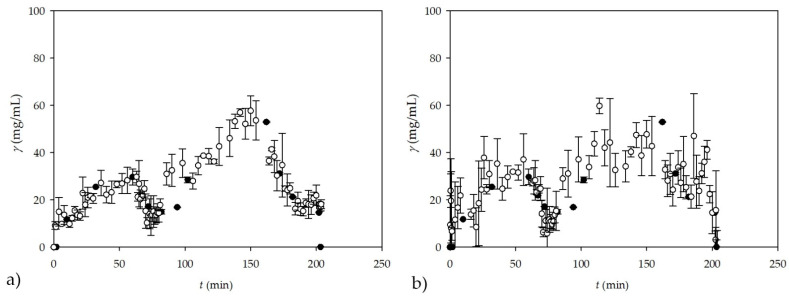
*On-line* measurement of glycerol concentration prediction by (**a**) ANN based on NIR spectroscopy for experiments performed in a +-shape microreactor for different residence times and (**b**) subsampling ANN based on NIR spectroscopy for experiments performed in a +-shape microreactor for different residence times (●—theoretical glycerol calculated from experimental data, ○—ANN predictions).

The second experiment, evaluating the ANN prediction of the NIR spectra, was performed with different residence times, which were changed frequently, before the steady state was established. The same principle as in the previous experiment was applied to find an ANN suitable for explaining the calculated theoretical values of the glycerol data. In this experiment, 90 samples were recorded in triplicate, resulting in a total of 270 samples. On GC, the glycerol concentration was measured for 51 samples, leaving 219 samples that were recorded by NIR spectroscopy only. As in the previous cases, PCA was first performed for all samples before ANN modeling. Again, ANN was performed for samples with known glycerol concentrations, which was used to predict other concentrations recorded by NIR spectroscopy only. The result was an ANN that had a separation of data into training, testing, and validation in ratio of 60:20:20, respectively. This time, the ANN consisted of 10 input layers (the first 10 PCA factors), 10 neurons in the hidden layer, and one output layer (glycerol concentration), with *R*^2^ values of 0.9719, 0.9733, and 0.9525 for training, testing, and validation, respectively. The results of ANN predictions of glycerol concentration during frequent changes of residence times are presented in Figure 10a. As in previous experiments, there is a clear agreement between *off-line* and *on-line* measurements, regardless of the different sampling times. From the Figure 10b it is visible that, once again, when working with some sort of cross-validation, results vary a bit from Figure 10a. Again, a subsampling strategy with five subsamples and a subsample size ratio of 60:20:20 was used with, in this case, 10 neurons in the hidden layer, and *R*^2^ values of 0.9785, 0.9574, and 0.9221 were obtained with errors of 0.0041, 0.0158, and 0.0217 for training, testing, and validation, respectively.

As for the previous experiment, where NIR spectra were also recorded in triplicate, a good trend of ANN predictions based on experimentally measured data is observed. It is also noticeable that this experiment was more dynamic as the residence times were changed frequently before the steady state was achieved. Although the ANN predictions for this experiment prove that NIR spectroscopy could be used as a potential *on-line* measurement method, a lot of work is still needed. Most importantly, a database of NIR spectra and GC measurements for all steady states needs to be created for this type of experiment to predict each transition state and glycerol concentration during that period. Nevertheless, in further experiments, additional data should be generated, which will enable the prediction of glycerol concentrations during any change in flow regime in a microreactor. Furthermore, as demonstrated in the work of Westad and Marini [37], some sort of cross-validation is needed when working with this kind of data; especially with ANNs that have less than a few hundreds or thousands of samples. As presented in this work, there is a clear difference between randomly selecting ANNs that had the highest *R*^2^ values and some kind of cross-validation that is needed in this case.

## 4. Conclusions

In this study, NIR spectroscopy was tested as a potential tool for monitoring the biodiesel production process. The results have shown that starting from the calibration of glycerol concentrations based on NIR spectra, and later for batch and microreactor processes where measurements were conducted, *off-line* predictions by ANN from NIR spectra gave results confirming that biodiesel production can be monitored by NIR spectroscopy. Additionally, in experiments where biodiesel production was monitored *on-line*, based on prediction models obtained from NIR measurements, NIR proves its potential for *on-line* monitoring of biodiesel production, both in a batch reactor and in continuous microreactor systems. However, when using the method, one should consider some of the disadvantages of ANNs, such as hardware dependency, i.e., ANNs need strong processors to work, and ANNs can give inexplicable results; therefore, proper network structure should be ensured and this requires good knowledge of working with various software packages. Moreover, the ANN algorithm can be unstable when working with a small number of samples, but once these drawbacks are overcome, one obtains a method that is fast, cheap, reliable, simple to perform, and repeatable.

## Figures and Tables

**Table 1:** Characteristics of ANN for predicting glycerol concentration.

Number	Network Configuration	Training	Training Error	Test	Test Error	Validation	Validation Error
1.	10-13-1	0.9996	0.0000	0.9997	0.0001	0.9961	0.0006
2.	10-9-1	0.9999	0.0000	0.9989	0.0001	0.9912	0.0024
3.	10-6-1	0.9998	0.0000	0.9996	0.0000	0.9988	0.0002
4.	10-8-1	0.9997	0.0000	0.9997	0.0001	0.9980	0.0003
5.	10-7-1	0.9995	0.0001	0.9992	0.0001	0.9959	0.0007
